# Simultaneous Qualitative Assessment and Quantitative Analysis of Metabolites (Phenolics, Nucleosides and Amino Acids) from the Roots of Fresh *Gastrodia elata* Using UPLC-ESI-Triple Quadrupole Ion MS and ESI- Linear Ion Trap High-Resolution MS

**DOI:** 10.1371/journal.pone.0150647

**Published:** 2016-03-08

**Authors:** Sha Chen, Jun Qiu Liu, Hui Xiao, Jun Zhang, An Liu

**Affiliations:** Key laboratory of Beijing for identification and safety evaluation of Chinese medicine, Institute of Chinese Materia Medica, China Academy of Chinese Medical Sciences, No. 16, Nanxiaojie, Dongzhimennei, Beijing, China; Macau University of Science and Technology, MACAO

## Abstract

A sensitive, effective and optimized method, based on ultra performance liquid chromatography (UPLC) coupled with ESI-triple quadrupole ion MS and ESI-linear ion trap high-resolution MS, has been developed for the simultaneous quantitative and qualitative determination of phenolics, nucleosides and amino acids in the roots of fresh *Gastrodia elata*. Optimization of the analytical method provided higher separation efficiency and better peak resolution for the targeted compounds. The simultaneous separation protocols were also optimized by routinely using accurate mass measurements, within 5 ppm error, for each molecular ion and the subsequent fragment ions. In total, 31 compounds, including 23 phenolics, two nucleosides, four amino acids, one gastrodin and one other compound were identified or tentatively characterized. Mono-substituted parishin glucoside (9), methoxy mono-substituted parishin (13), methyl parishin (26), *p*-hydroxybenzyl di-substituted parishin (29), and *p*-hydroxybenzyl parishin (31) were tentatively identified as new compounds. Principal metabolite content analysis and the composition of eight representative *G*. *elata* cultivars of various species indicated that geographic insulation was the main contributor to clustering.

## Introduction

*Gastrodia elata* (*G*. *elata*) is frequently used in traditional Chinese medicine (TCMs). TCMs made from root extractsare mainly used to treat vertigo, blackouts, headaches, dizziness, rheumatism, hemiplegia, and epileptic convulsions [1–3]. Many bioactive compounds have been isolated from *G*. *elata*, which include phenolics (4-hydroxybenzaldehyde, 4-hydroxybenzyl alcohol, gastrodin, and parishin), citric acid, and amino acids [4,5]. In recent years, the use of *G*. *elata* has increased rapidly as commercial preparations have become more readily available [6]. The compounds found in *G*. *elata* have been reported to possess anti-epileptic [7], anti-obesity [8], anti-convulsive [9], anti-oxidative [10] and memory enhancing properties [11].

The pharmacological properties of *G*. *elata* extracts are largely attributable to the presence of accumulated secondary metabolites. Although metabolite studies have, so far, focused mainly on the medicinal properties of *G*. *elata* products and extracts, a metabolite survey could also provide information about the genetic and biochemical control of the metabolism during the plant’s development. Additionally, *G*. *elata* may provide an interesting model for studying other biological processes and metabolic regulation [12–14].

The most abundant compound in *G*. *elata*, and also the main active ingredient, is parishin, an ester formed by the condensation of three gastrodin subunits. However, the *Chinese Pharmacopoeia* [15] designates parishin as the only characteristic ingredient of *G*. *elata*. Recently, though, many more compounds have been identified in *G*. *elata* by using mass spectrometry (MS). In one study [16], some parishin compounds that would be expected to occur in *G*. *elata* were not identified, and only m/z values of those identified in MS process were presented. It was challenging to make progress in the analysis of *G*. *elata* compounds. Furthermore, little information is available about the composition and content of metabolites in different cultivars of *G*. *elata*, as well as the relationship between the *G*. *elata* cultivars and their area of origin. A survey of these cultivars and their areas of orgin would therefore be valuable.

In addition to the above issues, separation efficiency and resolution of target peaks of compounds extracted from plant are common bottlenecks in the qualitative and quantitative analysis of metabolism [17]. If suitable separation protocols can't be established, the use of inappropriate protocols may influence the results of an analysis. The purpose of the present study was to develop a complete systematic method for the qualitative and quantitative analysis of the major bioactive compounds extracted from roots of *G*. *elata* by using ultra performance liquid chromatography (UPLC) coupled with ESI-triple quadrupole ion MS and ESI-linear ion trap high-resolution MS. To the best of our knowledge, this is a report describing a simple and time-saving analytical method for the simultaneous determination of multiple metabolites in fresh root samples from *G*. *elata*.

## Experimental

The study was carried out on private land and we confirmed that the owner of the land gave us the permission to conduct the study on this site.

### Chemicals and standards

HPLC grade acetonitrile used for HPLC-MS analysis, including acetonitrile and formic acid, were of HPLC grade (CNW, Dusseldorf, Germany). Formic acid was HPLC-grade obtained from Sigma-Aldrich (St. Louis, MO, USA). Waters used in the experiment was de-ionized and further purified by a Milli-Q Plus water purification system (Millipore Ltd.). Other reagents and chemicals were of analytical grade purchased from Beijing Chemistry factory Corporation. Gastrodin, parishin E, parishin B, and parishin were purchased from the National Institute for the control of Biological and Pharmaceutical Drugs (Beijing, China).

### Plant materials

Rhizomes from eight *G*. *elata* cultivars were collected from three independent populations; each population contained 60 individual plants. The eight cultivars were of four species (green, red, black, and hybrid) obtained from four representative provinces (Table 1). Three replicates of eight cultivars were manually collected in late December (12/24, 2013) from three individual plants of each cultivars. The collected rhizomes specimens were identified as *Gastrodia elata* Bl by a taxonomist (Professor Ming Cheng) at Institute of Chinese Materia Medica, China Academy of Chinese Medical Sciences, Beijing, China.

**Table 1:** *G*. *elata* cultivars used in this study.

Cultivars	Genotype groups	Place of collection (City-Province)	Coordinates (Latitude-Longitude)	Time of collection
1	Red#	Bijie-Guizhou	27°18'-105°18'	December 21, 2013
2	Green#	Bijie-Guizhou	27°18'-105°18'	December 21, 2013
3	Black#	Bijie-Guizhou	27°18'-105°18'	December 21, 2013
4	Red#	Yaan-Sichuan	29°59'-103°01'	December 22, 2013
5	Green#	Yaan-Sichuan	29°59'-103°01'	December 22, 2013
6	Hybrid#	Yaan-Sichuan	29°59'-103°01'	December 22, 2013
7	Red#	Hanzhong-Shanxi	33°04'-107°01'	December 24, 2013
8	Hybrid#	Zhaotong-Yunnan	27°20'-103°43'	December 25, 2013

### Sample preparation

For each freeze-dried sample of *G*. *elata* root, a portion (0.5 g, 100 mesh) was accurately weighed into a 50 mL flask and extracted with 20 mL 50% aqueous methanol (methanol: water 50:50, v/v) in an ultrasonic bath for 30 min at room temperature. Each extract combination was performed in triplicate. The extract was centrifuged for 5 min at 15, 000 g, the supernatant was collected and all the samples were re-extracted as above twice more. The combined supernatant was then filtered through a 0.22-μm Millipore filter (Alltech Scientific Corporation, Beijing, China) before injection for LC/MS analysis.

### UPLC method

Chromatographic separation was performed using a UPLC system (DaianU3000, Dionex Corparation, CA, USA). The equipment comprised an UPLC pump, a photodiode array (PDA) detector, and an auto-sampler set at 30°C. Phenolic detection in diode array detector was carried out at 270 nm, and spectrum scans were made from 200 to 400 nm. Separations were carried out using an Accucore C_18_ column (2.1 mm × 100 mm, 2.6 μm particle size, Thermo Fisher Scientific, Bellefonte, PA, USA). The mobile phase consisted of water containing 0.1% formic acid (A) and acetonitrile (B). Linear gradient elution was performed at a flow rate of 0.2 mL/min. The solvent gradient was changed according to the following program: 0–8 min, 2% B; 8–12 min, 2–8% B; 12–25 min, 8–12% B; and 25–47 min, 12–25% B. The injection volume was 1 μL and chromatograms were acquired at 270 nm.

### Q-Trap MS

MS analysis was performed using an LTQ Orbitrap mass spectrometer (Thermo Fisher Scientific, San Jose, CA, USA), fitted with an electrospray ionization (ESI) source operated in both negative and positive modes. The m/z range was 100–1200, with resolution set at 30000 using the normal scan rate. Data-dependent MS/MS events were always performed on the most intense ions detected in the full scan MS. The normalized collision energy was 30% for all compounds. Nitrogen was used as the sheath gas and helium as the collision gas. The key optimized ESI parameters were as follows: source voltage, 3.0 kV; sheath gas (nitrogen), 50 L/min; auxiliary gas flow, 10 L/min; capillary voltage, -35.0 V; capillary temperature, 350°C, and tube lens, -110.0 V. The ion injection time used was 50.0 ms. MS scan functions and UPLC solvent gradients were controlled by an X-calibur data system (Thermo Fisher, Scan Jose, CA, USA).

### ESI-QQQ-MS

Triple quadrupole (QQQ) scans were acquired using an Agilent 1290 UPLC-photodiode array 6460 triple quadrupole mass spectrometry system (Agilent Technologies, Palo Alto, CA, USA). The ESI source operation parameters were as follows: HV voltage, 3.5 kV; capillary, 7 μA; nozzle voltage, 500 V; delta emv, 300 V; gas flow, 5 L/min; gas temp, 350°C; nebulizer, 45 psi; sheath gas temp, 350°C; and sheath gas flow, 11 L/min.

### Method validation

The method was validated by characteristic indexes including linearity, the limit of detection (LOD), the limit of quantification (LOQ) and precision (inter-day. intra-day precision).

### Statistical analysis

Quantitative data were analyzed with SPSS 16.0 for window. Principal component analyses (PCA) for identifying homogeneous groups of individual tissues based on measured phenolics, nucleosides and amino acids concentrations were performed in SPSS 16.0. Factors were identified using varimax rotation and the two most significant factors were extracted using the Kaiser-Meyer-Olkin criterio (KMO).

## Results and Discussion

### Optimization of HPLC and MS conditions

**Fig 1:**
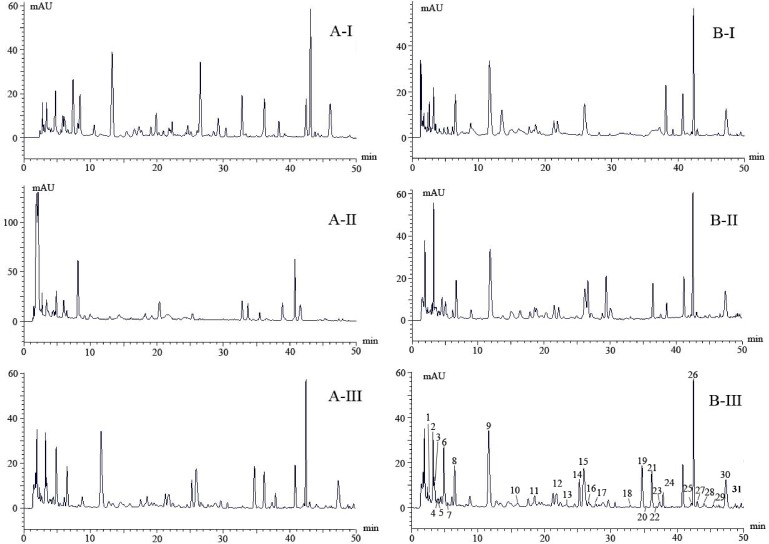
Optimized HPLC chromatograms at 220 nm selected from three column system (AI—AIII) and three mobile protocols (BI-BIII). Thirty-one compounds (peaks 1–31) were separated by BIII and identified using UPLC- LTQ Orbitrap mass spectrometry.

In preliminary tests, three columns were evaluated for the separation of target compounds from the roots of *G*. *elata*. These were a SunFire C_18_ column (2.1 mm × 100 mm, 1.9 μm particle size, Waters Corporation, Milford, USA) (Fig 1A-I), an Acquity BEH C_18_ column (2.1 mm × 100 mm, 1.7 μm particle size, Waters Corporation, Milford, USA) (Fig 1A-II), and an Accucore C_18_ column (2.1 mm × 100 mm, 2.6 μm particle size, Thermo Scientific, USA) (Fig 1A-III). Using the same elution protocol, the Accucore C_18_ column (Fig 1A-III) gave slightly better resolution than the other two columns. Thus, it indicated that particle size may be an important factor affecting the resolution of compounds in *G*. *elata*. Based on earlier reports, two elution systems (Fig 1B-I and 1B-II) were then evaluated to optimize the peak shapes of metabolites separated from *G*. *elata*. Elution system B-I, in which acetic acid was added to the eluent, was based on research carried out by Wang [18], and allowed identification of 15 phenolics and 6 nucleoside derivatives in the roots of *G*. *elata*. Elution system B-II, in which 0.1% formic acid was added to the mobile phase, was based on research carried out by Ong et al. [19] and has been widely used to separate phenolics from the roots of *G*. *elata*. In the present study, we found that a higher concentration of formic acid (0.5%, v:v, B-III) improved peak separation efficiency (Fig 1B-III). Our research suggests that an Accucore C_18_ column and a mobile phase containing formic acid (0.5%) is suitable for the analysis of metabolites in *G*. *elata*. The optimized experiment condition was replicated for eight different cultivars analysis.

### Method validation

#### Calibration curves, limits of detection and quantification

Method validation was carried out using four external standards (gastrodin, parishin E, parishin B, and parishin). The calibration curves showed good linearity for all standards at 350 nm (*r*^2^ ≥ 0.9915). The standard solutions were detected by chromatography until the signal-to-noise (S/N) ratios were 3 and 10; the corresponding concentrations at these S/N ratios were defined as the LOD and LOQ, respectively. The lowest and highest LOD and LOQ were obtained for gastrodin (0.024 and 0.081 μg/mL) and parishin (0.359 and 1.196 μg/mL) (Table 2).

**Table 2:** The regression equation, LOD and LOQ of the four standards using the optimized method for calibration.

Compounds	Regression equation	*r*^2^	Linear range (μg/mL)	LOD (μg/mL)	LOQ (μg/mL)
Gastrodin	*y* = 79899*x* - 1108.1	0.9999	8.89–44.48	0.024	0.081
Parishin E	*y* = 19656*x* - 12538	0.9915	6.08–60.80	0.143	0.475
Parishin B	*y* = 19656*x* - 12538	0.9981	34.50–138.00	0.104	0.345
Parishin	*y* = 315580*x* - 7052.4	0.9999	40.66–203.30	0.359	1.196

*y*, peak area; *x*, compound concentration (μg/mL); LOD = limit of detection, S/N = 3; LOQ = limit of quantitation, S/N = 10

#### Precision and accuracy of quantification

The precision of metabolite quantification was studied by examining the repeatability and intermediate precision for all compounds separated from *G*. *elata* roots. Six standard samples were evaluated on the same day to determine the intra-day precision. Three samples were also extracted and analyzed on three consecutive days to determine the inter-day precision. Sample solutions were prepared at three concentrations (low, middle, and high), with three replicates of each concentration in order to validate method precision. Relative standard deviations (RSDs) were calculated to assess repeatability and precision. The RSDs of the four compounds were less than 4.05% for inter-day precision at the three concentration and 4.00% for the intra-day precision (Table 3). The low RSD values obtained for the four compounds confirmed the high repeatability and intermediate precision of the method developed here.

**Table 3:** Intra- and inter-day precision of four standards by HPLC.

Compounds	Intra-day(n = 6)		Inter-day(n = 3)	
	Concentration (μg/mL)	RSD (%)	Concentration (μg/mL)	RSD ^b^ (%)
Gastrodin	9.48 ± 0.38 ^a^	4.00	9.30 ± 0.23	2.46
	15.85 ± 0.59	3.72	15.15 ± 0.30	2.01
	27.09 ± 0.34	1.25	27.09 ± 0.45	1.34
Parishin H	6.40 ± 0.21	3.35	6.29 ± 0.15	2.42
	8.99 ± 0.23	2.72	8.98 ± 0.15	1.48
	20.58 ± 0.66	3.18	20.02 ± 0.81	4.05
Parishin B	35.82 ± 0.59	1.67	35.76 ± 0.67	1.87
	57.56 ± 0.74	1.28	59.52 ± 0.78	1.30
	118.34 ± 0.60	0.51	118.39 ± 0.66	0.21
Parishin	50.88 ± 0.85	1.66	51.77 ± 0.41	0.80
	83.80 ± 0.61	0.72	84.08 ± 0.27	0.32
	167.23 ± 0.94	0.56	167.90 ± 0.81	0.48

^a^ Mean concentration ± SD.

^b^ RSD = (SD/mean) × 100

#### Accuracy and recovery of quantification

The accuracy of the method was investigated by measuring the recovery. This was assessed by adding three concentrations (high, middle and low) of standard solutions to known amounts of sample solution which were then extracted and subjected to quantitative analysis as described above. Each standard was tested at each concentration in triplicate. The equation used to define the percentage recovery was (detected amount—original amount)/spiked amount x 100. Recoveries obtained in this study were in the range 88.02–105.38% (Table 4), demonstrating that the analytical method developed in this study has high accuracy. The low RSDs of all standards (< 1.54%) indicate good reproducibility.

**Table 4:** Recovery of four standards in the extraction of GE (n = 3).

Compounds	Initial amount (mg)	Added amount (mg)	Total recovered amount^a^ (mg)	Recovery^b^ (%)	RSD^c^ (%)
Gastrodin	1.09	0.62	1.62 ± 0.01	88.02	1.26
	1.09	2.24	3.35 ± 0.03	100.91	1.54
	1.09	3.86	4.93 ± 0.01	99.65	0.30
Parishin E	1.22	0.42	1.62 ± 0.02	94.65	0.57
	1.22	2.04	3.33 ± 0.03	105.38	1.46
	1.22	4.11	5.01 ± 0.03	92.15	0.70
Parishin B	1.14	0.32	1.43 ± 0.00	89.88	0.52
	1.14	1.72	2.88 ± 0.01	101.96	0.58
	1.14	3.45	4.35 ± 0.00	92.85	0.13
Parishin	2.55	2.87	5.34 ± 0.01	97.66	0.34
	2.55	8.23	10.78 ± 0.02	100.43	0.00
	2.55	13.86	16.21 ± 0.01	98.49	0.07

^a^ Total recovered amount = mean content ± SD.

^b^ Recovery (%) = (detected amount—original amount)/spiked amount × 100.

^c^ RSD = (recovery SD/mean) × 100

### Analysis of constituents in *G*. *elata*

The identification of 31 compounds was performed in both positive and negative modes. Chemical structures, mass spectra (in PI, NI and NI-MS/MS), retention times, UV-Vis spectra, and retention times on the C_18_ column are listed in Table 5. The identified compounds could be classified into three groups, phenolics, nucleosides and amino acids, based on their chemical structures.

**Table 5:** Characterization of constituents extracted from gastrodia by ESI- linear ion trap high-resolution MS.

No.	Rt (min)	NI^−^		PI^+^			λmax (nm)	Identification
		[M-H]^−^, [M+HCOO]^−^	Fragment ions	[M+H]^+^, [M+Na]+	Fragment ions	ppm		
1	2.44	191.0198	111.10[M-H-2H_2_O-CO_2_]^−^, 173.10[M-H-H_2_O]^−^	215.0160	197.10[M+Na-H_2_O]^+^	—	256	Citric Acid
2	3.17	243.0123	—	267.0196	113.10[M+H-132]^+^	—	262	Uridine
3	3.51	180.1078	—	182.1056	165.10[M+H-NH_3_]^+^, 136.10[M+H-HCOOH]^+^	0.987	220	Tyrosine
4	3.74	—	—	132.1020	115.10[M+H-NH_3_]^+^, 86.10[M+H-HCOOH]^+^	0.721	217	Leucine
5	4.82	266.1567	134.12[M-H-132]^−^	268.1031	136.10[M+H-132]^+^	1.344	259	Adenosine
6	6.36	331.1044	161.12[Glu-H]^−^, 123.12[M-Glu-H]^−^	309.0925	185.10[Glu+Na]^+^	0.537	220	Gastrodin
7	8.12	—	—	127.1091	108.10[M+H- H_2_O]^−+^	—	285	5-hydroxymethyl fural
8	11.37	123.1098	105.12[M-H- H_2_O]^−^	—	—	0.503	220	P-hydroxybenzyl alcohol
9	21.83	621.1660	441.10[M-H-162-H_2_O]^−^, 459.10[M-H-162]^−^, 397.10[M-H-162-H_2_O-CO_2_]^−^, 369.10[M-H-162-CO_2_]^−^	645.1136	539.10[M+Na-106]^+^, 483.10[M+Na-162]^+^, 377.10[M+Na-268]^+^, 215.10[M+Na-268-162]^+^	0.411	222	Mono-substituted parishin glucoside
10	23.25	459.1145	173.10[M-H-268-H_2_O]^−^, 129.10[M-H-268-H_2_O-CO_2_]^−^	483.1123	377.11[M+Na-106]^+^, 321.11[M+Na-162]^+^, 215.11[M+Na -268]^+^, 185.11[Glu+Na]^+^	0.493	221	parishin H
11	25.15	459.1145	173.10[M-H-268-H_2_O]^−^, 129.10[M-H-268-H_2_O-CO_2_]^−^	483.1091	377.10[M+Na-2CO_2_-H_2_O]^+^, 321.10[M+Na-162]^+^, 215.10[M+Na -268]^+^, 185.10[Glu+Na]^+^	0.679	221	parishin E
12	25.72	412.1178	306.10[M-H-106]^−^	414.1331	339.10[M+H-75]^+^, 308.10[M+H-106]^+^, 285.12[M+H-147+H_2_O]^+^, 179.12[M+H-147+H_2_O-106]^+^, 233.12[M+H-106-75]^+^	2.094	224	S-(4-hydroxybenzyl) -glutathione
13	27.94	489.1247	427.10[M-H-H_2_O-CO_2_]^−^, 173.10[M-H-268-H_2_O-30]^−^	513.1048	377.00[M+Na-2Co_2_-H_2_O]^+^, 215.10[M+Na-268-30]^+^	0.436	223	Methoxy mono-substituted parishin
14	30.82	518.1593	412.10[M-H-162-106]^−^, 306.10[M-H-162-106-106]^−^	520.3333	377.10[M+Na-2CO_2_-H_2_O-30]^+^, 339.10[M+H-75-106]^+^, 308.10[M+H-106-106]^+^, 285.10[M+H-147+H_2_O-106]^+^, 233.10[M+H-106-75-106]^+^, 179.10[M+H-147+H_2_O-106-106]^+^	0.458	224	P-hydroxybenzyl s-(4-hydroxybenzyl) -glutathione
15	32.91	889.2617	621.10[M-H-268]^−^, 603.10[M-H-268-H_2_O]^−^, 585.10[M-H-268-2H_2_O]^−^, 559.10[M-H-268-H_2_O-CO_2_]^−^, 531.10[M-3H-268-2CO_2_]^−^, 423.10[M-H-268-2H_2_O-162]^−^, 397.10[M-H-268-H_2_O-CO_2_-162]^−^	913.2527	807.11[M+Na-106]^+^, 645.11[M+Na-268]^+^, 483.11[M+Na-268-162]^+^	—	224	Di-substituted parishin glucoside
16	35.50	727.2083	471.10[M-H-268-H_2_O]^−^, 453.10[M-H-268-2H_2_O]^−^, 423.10[M-H-268-2H_2_O-30]^−^, 397.10[M-H-268-H_2_O-CO_2_]^−^, 369.10[M-3H-268-2CO_2_-30]^−^	751.2071	645.10[M+Na-106]^+^, 483.10[M+Na-268]^+^, 539.10[M+Na-106-106]^+^, 589.10[M+Na-162]^+^, 377.10[M+Na-106-268]^+^, 215.10[M+Na-268-268]^+^	0.582	222	Di-substituted parishin
17	36.13	727.2092	459.10[M-H-268]^−^, 441.10[M-H-268-H_2_O]^−^, 423.10[M-H-268-2H_2_O]^−^, 397.10[M-H-268-H_2_O-CO_2_]^−^, 369.10[M-3H-268-2CO_2_]^−^	751.4514	645.10[M+Na-106]^+^, 483.10[M+Na-268]^+^, 539.10[M+Na-106-106]^+^, 589.10[M+Na-162]^+^, 377.10[M+Na-106-268]^+^, 215.10[M+Na-268-268]^+^	0.692	222	parishin B
18	36.22	889.2617	621.10[M-H-268]^−^, 603.10[M-H-268-H_2_O]^−^, 585.10[M-H-268-2H_2_O]^−^, 559.10[M-H-268-H_2_O-CO_2_]^−^, 531.10[M-3H-268-2CO_2_]^−^, 423.10[M-H-268-2H_2_O-162]^−^, 397.10[M-H-268-H_2_O-CO_2_-162]^−^	913.1057	807.10[M+Na-268+Glc]^+^, 645.10[M+Na-268]^+^, 483.10[M+Na-268-162]^+^	—	224	Di-substituted parishin glucoside isomer
19	37.26	757.2189	471.10[M-H-268-H_2_O]^−^, 453.10[M-H-268-2H_2_O]^−^, 423.11[M-H-268-2H_2_O-30]^−^, 397.10[M-H-268-H_2_O-CO_2_]^−^, 369.10[M-3H-268-2CO_2_-30]^−^	781.4503	483.10[M+Na-268-162-30]^+^, 513.10[M+Na-268]^+^, 675.10[M+Na-268+Glu]^+^	5.178	225	Methoxy di-substituted parishin
20	37.98	727PONE-D-15-37678R3.2070	459.10[M-H-268]^−^, 441.10[M-H-268-H_2_O]^−^, 423.10[M-H-268-2H_2_O]^−^, 397.10[M-H-268-H_2_O-CO_2_]^−^, 369.10[M-3H-268-2CO_2_]^−^	751.2071	645.10[M+Na-106]^+^, 483.10[M+Na-268]^+^, 539.10[M+Na-106-106]^+^, 589.10[M+Na-162]^+^, 377.10[M+Na-106-268]^+^, 215.10[M+Na-268-268]^+^	3.049	222	parishin C
21	39.11	757.2189	471.10[M-H-268-H_2_O]^−^, 453.10[M-H-268-2H_2_O]^−^, 423.10[M-H-268-2H_2_O-30]^−^, 397.10[M-H-268-H_2_O-CO_2_]^−^, 369.10[M-3H-268-2CO_2_-30]^−^	781.4555	483.10[M+Na-268-162-30]^+^, 513.10[M+Na-268]^+^, 675.10[M+Na-268+Glu]^+^	5.178	225	Methoxy di-substituted parishin isomer
22	39.51	741.2240	741.10[M-H]^−^, 473.10[M-H-268]^−^, 441.10[M-H-268-H_2_O-14]^−^	765.2215	659.10[M+Na-106]^+^, 603.10[M+Na-162]^+^, 497.10[M+Na-268-14]^+^, 391.10[M+Na-106-268]^+^	0.550	224	Methyl di-substituted parishin
23	40.59	787.2303	741.10[M-H]^−^, 473.10[M-H-268]^−^, 441.10[M-H-268-H_2_O-14]^−^	765.1089	659.10[M+Na-106]^+^, 603.10[M+Na-162]^+^, 497.10[M+Na-268-14]^+^, 391.10[M+Na-106-268]^+^	0.610	224	Methyl di-substituted parishin isomer
24	42.02	1157.3546	889.10[M-H-268]^−^, 727.10[M-H-268-162]^−^, 585.10[M-H-2TMS]^−^, 423.10[M-H-2TMS-162]^−^, 379.10[M-H-2TMS-CO_2_-162]^−^	1181.1189	913.10[M+Na-TMS+H_2_O]^+^, 807.10[M+Na-268-268+Glu]^+^, 751.10[M+Na-268-162]^+^, 645.10[M+Na-268-268+Glu-162]^+^, 483.10[M+Na-268-268-162]^+^	3.271	223	Parishin glucoside
25	42.39	995.3034	727.10[M-H-268]^−^, 441.10[M-H-268-268-H_2_O]^−^, 423.10[M-H-268-268-2H_2_O]^−^, 397.10[M-H-268-268-H_2_O-CO_2_]^−^, 379.10[M-H-268-268-2H_2_O-CO_2_]^−^	1019.1771	913.10[M+Na-106]^+^, 857.10[M+Na-162]^+^, 807.10[M+Na-106-106]^+^, 751.10[M+Na-268]^+^, 645.10[M+Na-268-268+Glu]^+^, 589.10[M+Na-162-268]^+^, 539.10[M+Na-106-106-268]^+^, 483.10[M+Na-268-268]^+^, 377.10[M+Na-106-268-268]^+^	1.797	222	Parishin
26	42.81	1025.3138	757.10[M-H-268]^−^, 727.10[M-H-268-30]^−^	1049.3097	781.10[M+Na-268]^+^, 751.10[M+Na-268–30]^+^, 645.10[M+Na-268-268+162–30]^+^, 483.10[M+Na-268-268-30]^+^	0.760	224	Methyl parishin
27	43.01	565.1553	529.10[M-H-2H_2_O]^−^, 503.10[M-H-CO_2_-H_2_O]^−^, 459.10[M-H-106]^−^, 397.10[M-H-106-H_2_O-CO_2_]^−^, 173.10[M-H-268-H_2_O-106]^−^	589.1525	571.10[M+Na-H_2_O]^+^, 483.10[M+Na-106]^+^, 215.10[M+Na-268-106]^+^	0.091	224	P-hydroxybenzyl mono-substituted parishin
28	43.89	565.1556	529.10[M-H-2H_2_O]^−^, 503.10[M-H-CO_2_-H_2_O]^−^, 459.10[M-H-106]^−^, 397.10[M-H-106-H_2_O-CO_2_]^−^, 173.10[M-H-268-H_2_O-106]^−^	589.1523	571.10[M+Na-H_2_O]^+^, 483.10[M+Na-106]^+^, 215.10[M+Na-268-106]^+^	0.091	224	P-hydroxybenzyl mono-substituted parishin isomer
29	44.34	833.2500	727.10[M-H-106]^−^, 441.10[M-H-268-H_2_O-106]^−^, 397.10[M-H-268-H_2_O-CO_2_-106]^−^	857.2483	751.10[M+Na-106]^+^, 645.10[M+Na-268+162–106]^+^, 589.10[M+Na-268]^+^, 483.10[M+Na-268-106]^+^, 377.10[M+Na-268-106-106]^+^	0.893	228	P-hydroxybenzyl di-substituted parishin
30	45.91	833.2501	727.10[M-H-106]^−^, 441.10[M-H-268-H_2_O-106]^−^, 423.10[M-H-268-2H_2_O-106]^−^, 397.10[M-H-268-H_2_O-CO_2_-106]^−^, 369.10[M-3H-268-2CO_2_-106]^−^, 263.10[M-3H-268-2CO_2_-106-106]^−^	857.2461	751.10[M+Na-106]^+^, 645.10[M+Na-268+162–106]^+^, 589.10[M+Na-268]^+^, 483.10[M+Na-268-106]^+^, 377.10[M+Na-268-106-106]^+^	0.893	228	P-hydroxybenzyl di-substituted parishin isomer
31	46.18	1101.3436	995.10[M-H-106]^−^, 833.10[M-H-268]^−^, 727.10[M-H-268-106]^−^	1125.3408	1019.10[M+Na-106]^+^, 857.10[M+Na -268]^+^, 589.10[M+Na-268-268]^+^, 483[M+Na-268-268-106]^+^	0.984	225	P-hydroxybenzyl parishin

Mass spectra of metabolites from *G*. *elata* in both positive and negative mode showed that the characteristic ions originated mainly from functional groups such as hydroxyl and carboxyl groups. By comparing UPLC retention times and UV and mss spectral data with those of reference standards, the target peaks were tentatively identified as described below.

#### MS^n^ analysis of nucleosides in *G*. *elata*

In a previous reference, two nucleosides (peaks 2 and 5) that showed similar MS fragmentation patterns, including loss of a ribose moiety (132 Da), were identified in *G*. *elata* extracts. In positive ionization mode, nucleosides mainly showed molecular ions [M+Na]^+^; in negative ionization mode, abundant [M-H]^-^ or [M+HCOO]^-^ ions were observed. By comparison with literature data [18], peak 2 was tentatively identified as uridine. In positive mode, the MS/MS spectrum focused on the precursor ion [M+Na]^+^ (m/z 267.0196) showed a peak corresponding to loss of a ribose moiety (m/z 113, [M+H-132]^+^) (Table 5). Peak 5 was tentatively identified as adenosine, based on an earlier report [20]. Its fragmentation pattern was similar to that of uridine, with a characteristic fragment at m/z 136 [M+H-132]^+^.

#### Analysis of amino acids in *G*. *elata*

Although the UV absorption of the majority of amino acids is weak, the characteristic mass spectra of four amino acids were found in *G*. *elata* extracts.

Peak 3 produced an [M+H]^+^ ion at m/z 182.1056 and an [M-H]^-^ ion at m/z 180.1078, with fragment ions at m/z 165 [M+H-NH_3_] ^+^ and 136 [M+HCOOH]^+^. It can be identified as tyrosine based on a previous report [21]. Peak 4 showed an [M+H] ^+^ at m/z 132.1020 in positive ion mode and was identified as leucine. Product ions at m/z 115 [M+H-NH_3_]^+^ and 86 [M+H-HCOOH]^+^ were observed in the MS/MS spectrum. The MS data of peak 12 ([M+H]^+^ m/z 414.1331 and MS/MS m/z 285/179/233/) indicated that this compound contained m/z 285 [M+H-147+H_2_O]^+^, 179 [M+H-147+H_2_O-106]^+^ and 233 [M+H-106-75]^+^ groups. By comparison with an earlier report [22], peak 12 was identified as S-(4-hydroxybenzyl)-glutathione. Peak 14 showed the same behavior in the mass spectrum as peak 12 but with an additional 106 Da fragment, indicating a hydroxybenzyl linkage. MS-MS analysis of precursor ions at m/z 414 [M+H-106]^+^, m/z 339 [M+H-75-106]^+^, m/z 308 [M+H-106-106]^+^, m/z 285 [M+H-147+H_2_O-106]^+^, m/z 233 [M+H-106-75-]^+^, and m/z 179 [M+H-147+H_2_O-106-106]^+^ are characteristic of an amino linkage, and the compound was tentatively identified as *p*-hydroxybenzyl-S-(4-hydroxybenzyl)-glutathione.

#### MS^n^ analysis of phenolic in *G*. *elata*

**Fig 2:**
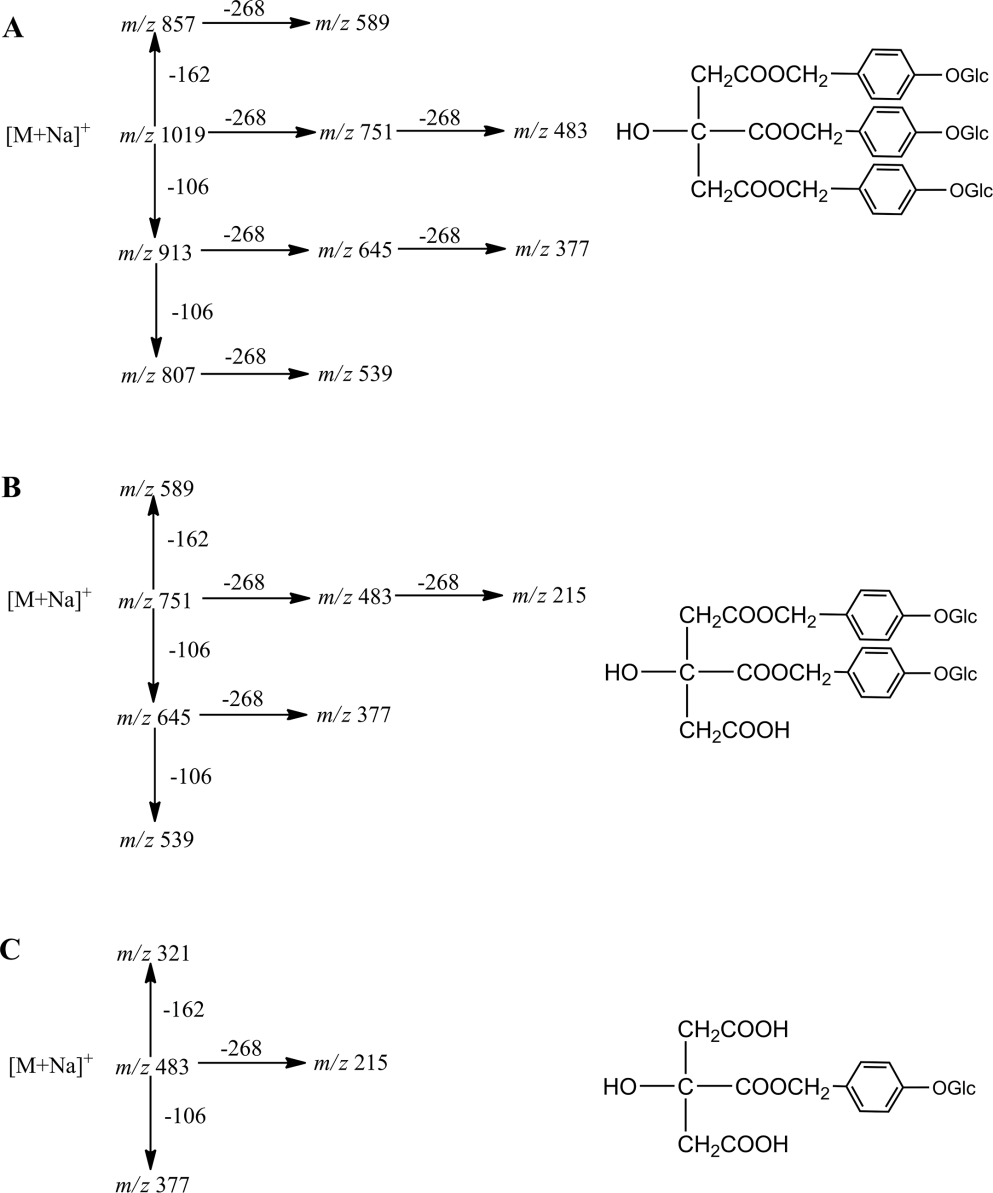
MS^n^ spectrum behavior of parishin (A), parishin B (B), parishin E (C) in *G*. *elata*.

The external standards, parishin, parishin B, and parishin E, were used to study one to third-order collision-induced dissociation (CID) spectra (MS^3^) for phenolic identification. Full confidence fragmentation pathways could be achieved progressively, following comparison of product ion mass spectra of the compounds under investigation with those recorded for parishin (Fig 2). Analyses of product ion spectra of molecules [M+Na]^+^ provided information about the size of substituents as well as substitution patterns of parishin analogues. Similarly, for di- and mono-glycosides of parishin derivatives (9, 13, 22, 26, 29, and 31), MS/MS spectra of the [M+Na]^+^ ions contained either [M+Na-162]^+^, [M+Na-106]^+^, [M+Na-268]^+^, [M+Na-162-268]^+^, or [M+Na-106-268]^+^ (As shown in S1 Fig) The six parishin derivatives were tentatively identified as described below.

Peak 9 showed an [M+Na]^+^ ion at m/z 645.1136, and MS/MS showed consecutive losses of 106 Da at m/z 539 [M+Na-106]^+^, 162 Da at m/z 483 [M+Na-162]^+^, 268 Da at m/z 377 [M+Na-268]^+^, and 430 Da at m/z 215 [M+Na-268-162]^+^ from the protonated molecule. The mass spectral behavior and molecular weight were confirmed in negative ion mode, with the deprotonated molecule [M-H]^-^ at m/z 621.1660 and MS/MS showing neutral losses of glucoside, H_2_O and CO_2_, at m/z 441 [M-H-162-H_2_O]^-^, 459 [M-H-162]^-^, 397 [M-H-162-H_2_O-CO_2_]^-^, and 369 [M-H-162-CO_2_]^-^. The characteristic neutral loss of a 162 Da fragment is typically observed for parishin glucosides and glucoside is also the only substituent previously reported in *G*. *elata* [18]. Peak 9 was thus tentatively identified as mono-substituted parishin glucoside.

Peak 13 was tentatively identified as methoxy mono-substituted parishin, based on similar mass spectral behavior to peak 17 (parishin B). Peak 13 had a MW of 490, with a deprotonated molecular ion [M-H]^-^ at m/z 489.1247 in negative ion mode. In positive ion mode, a molecular ion was observed at m/z 513.1048 [M+Na]^+^ and fragments were observed at m/z 414 [M+H-106]^+^ and 215 [M+Na-268-30]^+^ during the MS/MS process.

Peak 26 was tentatively identified as methyl parishin, MW 1026, with a deprotonated molecular ion [M-H]^-^ at m/z 1025.3138 in negative mode. In positive mode, an ion was observed at m/z 1049.3097 [M+Na]^+^, and MS^2^ showed neutral losses of 268 Da and 30 Da as well as characteristic ions at m/z 781 [M+Na-268]^+^, m/z 751 [M+Na-268-30]^+^, m/z 645 [M+Na-268-268-162-30]^+^, and 483 [M+Na -268-268-30]^+^, indicating that peak 26 is a methyl-substituted parishin.

The molecular weight of peak 29 was 834, as determined by the ions observed at m/z 833.2500 [M-H]^-^ and m/z 857.2483 [M+Na]^+^. MS/MS showed neutral loss of 106 (m/z 727 [M-H-106]^-^ and m/z 751 [M+Na-106]^+^), as well as m/z 589 [M+Na-268]^+^, m/z 483 [M+Na-268-106]^+^, and m/z 377 [M+Na-268-106-106]^+^ in positive mode. In negative mode, m/z 441 [M-H-268-H_2_O-106]^-^ and 397 [M-H-268-106-H_2_O-CO_2_]^-^ were obtained. These typical fragmentations were observed for peak 29, which was tentatively identified as *p*-hydroxybenzyl di-substituted parishin.

Peak 31 was tentatively identified as *p*-hydroxybenzyl parishin, MW 1102, with a deprotonated molecular ion [M-H]—at m/z 1101.3436 in negative mode and [M+Na] ^+^ at m/z 1125.3408 in positive mode. Characteristic neutral losses of 106 Da and 268 Da were observed in the MS/MS process. The identification was confirmed by fragments obtained from the ion at m/z 995 [M-H-106]^-^, 833 [M-H-268]^-^, and 727 [M-H-268-106]^-^_in negative mode, as well as ions at m/z 1019 [M+Na-106]^+^, 857 [M+Na-268]^+^, 751 [M+Na-268-268]^+^, and 483 [M+Na-268-268-106]^+^ in positive mode (Table 5).

Based on literature reports [10,18,20,23–26], retention times on the C_18_ column, UV-Vis spectra and co-elution with standards, the other peaks were identified as shown in Table 5.

Overall, thirty-one phenolic compounds were identified in fresh *G*. *elata* plant extracts in this study (Table 5). Using UPLC coupled with ESI-triple quadrupole ion MS and ESI-linear ion trap high-resolution MS, mono-substituted parishin glucoside (peak 9), methoxy mono-substituted parishin (peak 13), methyl parishin (peak 26), *p*-hydroxybenzyl di-substituted parishin (peak 29), *p*-hydroxybenzyl parishin (peak 31) were detected and identified for the first time in *G*. *elata*.

### Quantitative and qualitative analysis of compounds

Significant qualitative differences were found in the metabolites detected in different species as well as in plants collected from different producing areas. Interestingly, the characteristic compound, gastrodin, was not detected in the red *G*. *elata* cultivar collected from Shanxi province. S-(4-hydroxybenzyl)-glutathione was detected only in the hybrid obtained from Sichuan province.

Compounds gastrodin, parishin E, parishin B, parishin were quantified using their available counterparts as standards, and the other parishin derivative compounds using parishin as the standard, while other compounds using gastrin as the standard. all the studied 31 compounds were classified as five groups include amino acids, nucleosides, S-(4-hydroxybenzyl)-glutathione, gastrodin, parishin derivates for further statistical analysis.

### Principal component analyses

**Fig 3:**
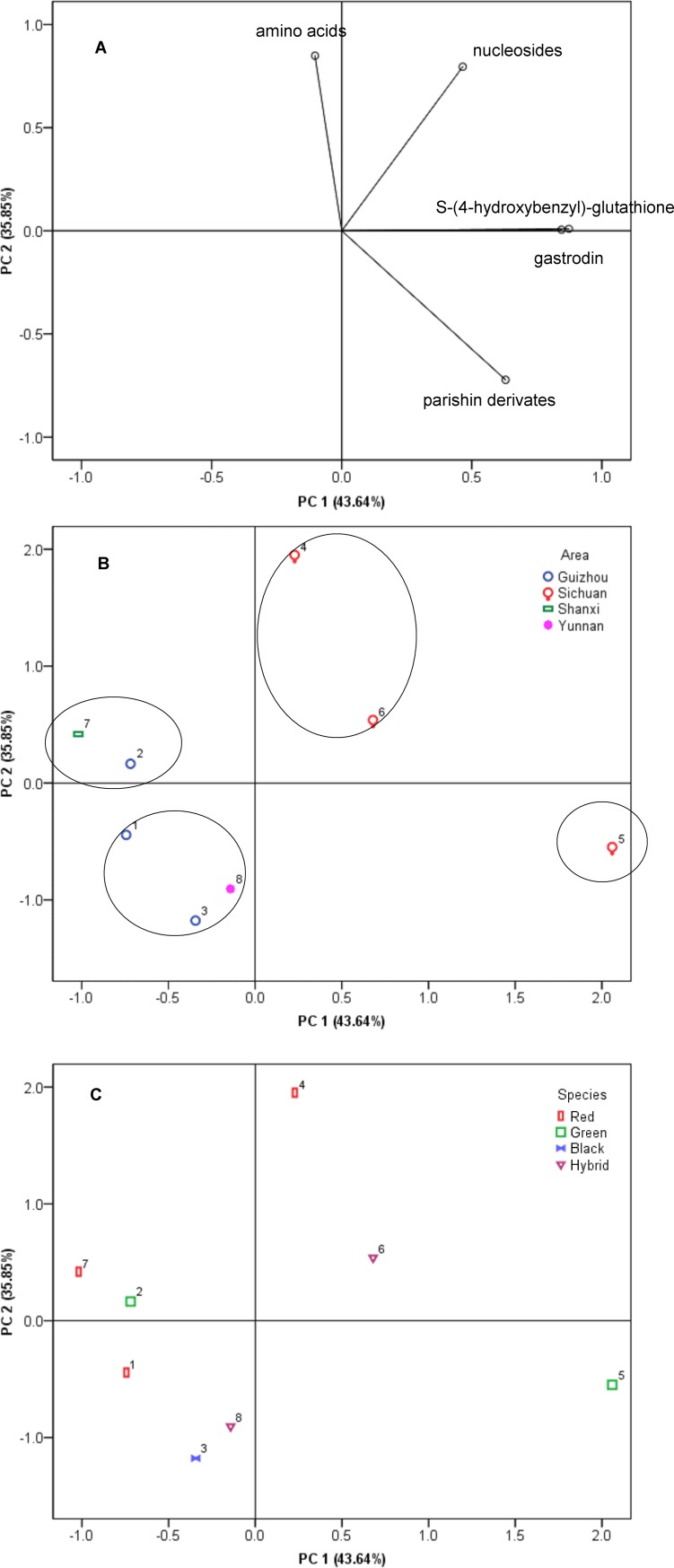
Positions of PCA scores (PC1, PC2) of eight *G*. *elata* cultivars, based on amino acids, nucleosides, S-(4-hydroxybenzyl)-glutathione, gastrodin, parishin derivatives compound content. The four compounds (gastrodin, parishin E, parishin B, parishin) were quantified by comparison with external standards, while the other parishin derivatives were quantified by comparison with an equivalent of parishin, and the other compounds were compared with an equivalent of gastrodin. Percentages in parentheses represent principal component variance. Numbers in figure B and C correspond to cultivar number in Table 1. (A) Sore scatter plot; (B) loading plot based on different area;(C)loading plot based on different cultivars. All the quantitative data was acquired by UPLC-ESI-triple quadrupole ion MS.

Principal component analysis (PCA) was used to provide an overview of the complete data set, showing variability between compounds detected and *G*. *elata* growth area or species. PCA using these attributes could explain 79.49% of the variance, partitioned as 43.64% in principal component 1 (PC 1) and 35.85% in principal component 2 (PC 2) (Fig 3). The loading of PC 1 showed strong positive correlations with S-(4-hydroxybenzyl)-glutathione, gastrodin, and parishin derivatives whereas PC 2 showed an important positive correlation with amino acids and nucleosides. Relationships between *G*. *elata* collection areas and detected compounds are shown in Fig 3A and Fig 3B and relationships between *G*. *elata* species and detected compounds are shown in Fig 3A and 3C.

As shown in Fig 3B, the eight *G*. *elata* cultivars could be divided into four groups. Group I, comprising two *G*. *elata* cultivars (4 and 6) collected from Sichuan province, was located to the right of the PC1 axis and above the PC2 axis. *G*. *elata* cultivars collected from Sichuan province were characterized by high levels of amino acids and nucleosides. Group II consisted of one cultivar (5) from Sichuan province and was characterized by high levels of S-(4-hydroxybenzyl)-glutathione and gastrodin. Group III was located in the second quadrant and contained cultivars 2 (Guizhou, *G*. *elata*) and 7 (Shanxi, *G*. *elata*,) which have relatively high levels of amino acids. Group IV, located in the lower left part of the scatter plot, included two *G*. *elata* samples obtained from Guizhou (1 and 3) and one *G*. *elata* sample obtained from Yunnan (8) and was characterized by very low individual compound content.

PCA demonstrated a lack of strong characteristic clustering among different *G*. *elata* species. To some extent, our results show that geographic insulation affects metabolite synthesis to a greater extent than species diversity.

## Conclusions

A reliable and effective method using UPLC coupled with ESI-triple quadrupole ion MS and ESI-linear ion trap high-resolution MS was successfully developed for online identification of low molecular weight metabolites of *G*. *elata*. A total of 31 compounds were identified or tentatively characterized, based mainly on fragment ion information obtained by UPLC-MS/MS. Five of these compounds were identified for the first time. PCA showed that the synthesis of *G*. *elata* metabolites varies with both species and geographic insulation. The analysis study of the active compounds in *G*. *elata* where quality control is of interest, in which case identification based on metabolites detection is possible and necessary.

## Supporting Information

S1 Fig MS/MS spectra of peak 9, peak 13, peak 26, peak 31. (JPG)
