# Supplementary figures and images for: Simultaneous Qualitative Assessment and Quantitative Analysis of Metabolites (Phenolics, Nucleosides and Amino Acids) from the Roots of Fresh Gastrodia elata Using UPLC-ESI-Triple Quadrupole Ion MS and ESI- Linear Ion Trap High-Resolution MS
Source: PLoS One. 2016 Mar 8;11(3):e0150647. doi: 10.1371/journal.pone.0150647 (PMC4783114; doi:10.1371/journal.pone.0150647)

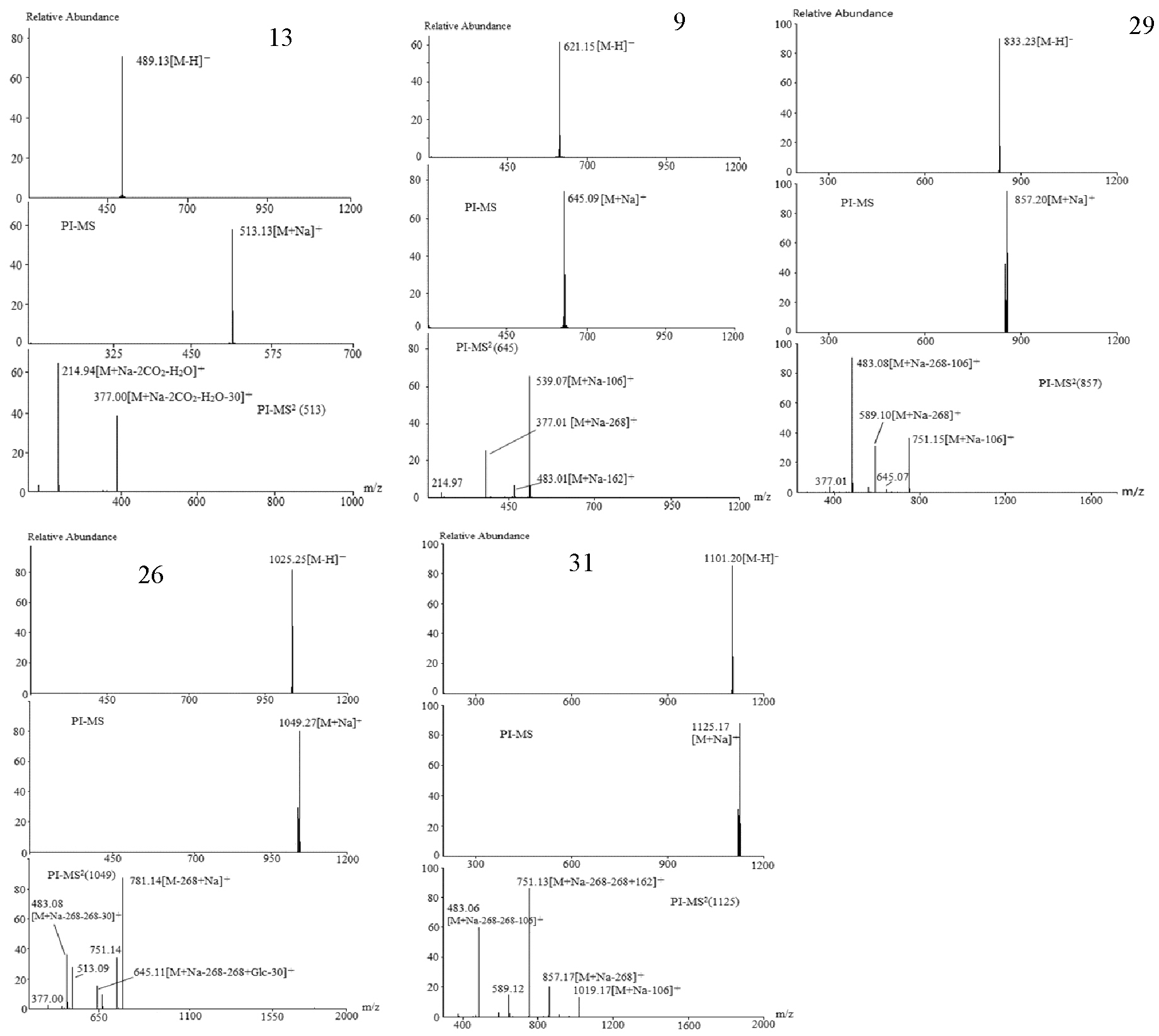

Supplement: S1 Fig — (JPG) [file pone.0150647.s001.JPG]
